# Imaging-Based Tumor Margin Patterns and Primary Tumor Categories and Their Association with Adverse Histopathological Features in Oral Tongue Squamous Cell Carcinoma

**DOI:** 10.3390/cancers18193118

**Published:** 2026-09-25

**Authors:** Nivedita Chakrabarty, Tapish Dadlani, Abhishek Mahajan, Arjun Singh, Swapnil Rane, Sarbani Ghosh Laskar, Ankush Shetake, Vanita Noronha, Kumar Prabhash, Pankaj Chaturvedi

**Affiliations:** 1 Department of Radiodiagnosis, Tata Memorial Centre, Advanced Centre for Treatment, Research and Education in Cancer (ACTREC), Kharghar, Navi Mumbai 410210, Maharashtra, India; 2 Homi Bhabha National Institute (HBNI), Training School Complex, Anushakti Nagar, Mumbai 400094, Maharashtra, India; 3 Department of Radiodiagnosis, Tata Memorial Hospital, Homi Bhabha National Institute (HBNI), Parel, Mumbai 400012, Maharashtra, India; tapishdadlani@gmail.com; 4 Department of Imaging, The Clatterbridge Cancer Centre NHS Foundation Trust, 65 Pembroke Place, Liverpool L7 8YA, UK; abhishek.mahajan@nhs.net; 5 Faculty of Health and Life Sciences, University of Liverpool, Liverpool L7 8TX, UK; 6 Department of Head and Neck Surgical Oncology, Tata Memorial Centre, Advanced Centre for Treatment, Research and Education in Cancer (ACTREC), Kharghar, Navi Mumbai 410210, Maharashtra, India; asingh@actrec.gov.in (A.S.); pankaj.chaturvedi@actrec.gov.in (P.C.); 7 Department of Pathology, Tata Memorial Centre, Advanced Centre for Treatment, Research and Education in Cancer (ACTREC), Kharghar, Navi Mumbai 410210, Maharashtra, India; srane@actrec.gov.in; 8 Department of Radiation Oncology, Tata Memorial Centre, Tata Memorial Hospital, Parel, Mumbai 400012, Maharashtra, India; laskarsg@tmc.gov.in; 9 Statistics, Department of Clinical Research Secretariat, Tata Memorial Centre, Tata Memorial Hospital, Parel, Mumbai 400012, Maharashtra, India; ankush.shetake@tmc.gov.in; 10 Department of Medical Oncology, Tata Memorial Centre, Tata Memorial Hospital, Parel, Mumbai 400012, Maharashtra, India; noronhavm@tmc.gov.in (V.N.); prabhashk@tmc.gov.in (K.P.)

**Keywords:** imaging-based tumor margin, oral tongue carcinoma, worst pattern of invasion

## Abstract

**Background:** This retrospective study introduces imaging inner tumor margin patterns (iITMP) and evaluates their correlation with pathological Worst pattern of invasion (WPOI) in oral tongue squamous cell carcinoma (OTSCC). Associations of iITMP with pathological primary tumor category (pT), histologic grade, perineural invasion (PNI), nodal metastasis, and extranodal extension (ENE) were assessed. Correlations between imaging depth of invasion (iDOI) and pathological DOI (pDOI), and between imaging primary tumor (iT) and pT, PNI, and ENE were evaluated. **Methods:** Baseline magnetic resonance imaging (MRI) from January 2021 to December 2022 of 224 upfront-operated OTSCC patients was reviewed by two blinded radiologists using contrast-enhanced thin-slice. iITMP (types 1–3) were classified based on inner tumor margin morphology. iDOI was measured after excluding peritumoral hyperenhancement. Correlation between categorical and continuous variables were assessed using Kappa statistics and Spearman’s test respectively. Associations between categorical variables were analyzed using Fisher’s exact or Pearson’s chi-squared tests. Univariable and multivariable analyses were additionally performed. **Results:** The mean patient age was 47 ± 11 years. Substantial agreement was observed between iITMP and WPOI, and between iT and pT. iDOI strongly correlated with pDOI. iITMP was significantly associated with pT, irregular (iITMP2) and infiltrative (iITMP3) margins were associated with PNI only on univariate analysis, while iITMP was not an independent predictor on multivariable analysis. iT significantly correlated with PNI and ENE. **Conclusions:** iITMP strongly correlate with WPOI and is substantially associated with pT categories. iT categories show significant association with PNI and ENE. Therefore, iITMP and iT may serve as potential imaging biomarkers of adverse histopathological features in OTSCC.

## Simple Summary

Magnetic resonance imaging plays an important role in the preoperative assessment of oral tongue squamous cell carcinoma, particularly for evaluating tumor extent and depth of invasion. However, the ability of magnetic resonance imaging to predict aggressive histopathological features remains less well established. In this study, we evaluated preoperative magnetic resonance imaging in 224 patients with oral tongue squamous cell carcinoma who underwent upfront surgery. We developed a simple imaging-based classification of the inner tumor margin into lobulated, irregular, and infiltrative patterns and assessed its relationship with pathological findings. The imaging-margin patterns showed substantial agreement with the pathological patterns of invasion and were significantly associated with pathological tumor stage. The imaging-based primary tumor categories showed significant association with perineural invasion. These findings suggest that detailed assessment of tumor morphology on magnetic resonance imaging may provide useful preoperative information about aggressive tumor behavior and assist in treatment planning.

## 1. Introduction

Oral cavity cancer is the 16th most common cancer in the world and the second most frequent cancer in India [1,2]. Tongue is one of the frequently involved subsites in oral cavity cancer and upfront surgery, with/without adjuvant therapy, remains the predominant management strategy for oral tongue squamous cell carcinoma (OTSCC) [3,4,5,6,7]. The ninth version of the Union for International Cancer Control (UICC) Tumor Node Metastasis (TNM) staging for head and neck cancers retains the oral cavity cancer staging from the eighth edition, with the added clarification that superficial extension from the mucosal lip to adjacent skin (such as the dry vermilion and vermilion border of the lip) alone does not constitute T4a disease [8,9]. Depth of invasion (DOI) is an integral component of oral cavity staging and knowledge of preoperative DOI on imaging helps in decision regarding elective neck dissection, extent of surgery and requirement for reconstruction, and planning adjuvant therapy [8,10,11]. Worst pattern of invasion (WPOI) scores of four and five on post-operative histopathology are associated with worse overall survival (OS), higher rates of recurrence, and increased likelihood of lymph node metastasis [4,12]. Studies have shown that perineural invasion (PNI) on post-operative histopathology is associated with worse OS, disease free survival (DFS), disease-specific survival (DSS), and with recurrence [13,14,15]. Extranodal extension (ENE) is a known poor prognostic factor included in the oral cavity staging and its presence requires adjuvant chemoradiotherapy (CRT) [8,16,17,18,19,20].

Primary objectives of our study were:

- To develop imaging inner tumor margin patterns (iITMP) and evaluate its correlation with pathological WPOI for OTSCC.
- To evaluate the association of iITMP with pathological primary tumor (pT) categories, histopathological grade of OTSCC, PNI, metastatic nodal involvement and ENE.
- To correlate imaging DOI (iDOI) with pDOI and evaluate category-wise correlation of imaging primary tumor (iT) with pT.

Secondary objectives of the study were to correlate iT with PNI and ENE for OTSCC.

## 2. Materials and Methods

### 2.1. Study Design and Study Population

It was a retrospective observational study in a tertiary cancer hospital. Baseline MRI of upfront operated patients with OTSCC from 1 January 2021 till 31 December 2022 were evaluated after obtaining Institutional Ethical Committee approval. A total of 224 patients who satisfied inclusion and exclusion criteria were enrolled. Post operative histopathological data was obtained from the hospital electronic medical records (EMR). 

Patients were included if a baseline (pre-treatment) MRI had been performed within four weeks of upfront surgery for OTSCC. The MRI was required to include T1-weighted imaging, T2-weighted imaging, short tau inversion recovery (STIR), and a contrast-enhanced (CE) Fast Spoiled Gradient Echo (FSPGR) sequence with a section thickness of 1.5 mm, all available in the picture archiving and communication system (PACS). Additionally, a post-operative histopathology report (HPR) was required to be available in the electronic medical record (EMR).

Patients were excluded if tumors were located outside the oral tongue, if treatment-naïve MRI images were unavailable, or if MRI image quality was inadequate. Exclusion was also applied if the post-operative HPR lacked details on WPOI, histopathological grade, pDOI, pT and pN categories, ENE, or PNI. Furthermore, patients were excluded if the histopathology was of a type other than squamous cell carcinoma. Figure 1 shows the flow diagram for selection of eligible patients.

### 2.2. Image Analysis

The MRI images available on PACS for all 224 patients were evaluated by two blinded radiologists with over 10 years and 5 years of experience in head and neck radiology, respectively, and any discrepancies were mutually resolved. MRI images were acquired either in General Electric (GE)-Signa explorer 1.5T or Philips-Ingenia 1.5T. All patients received 0.1 mmol/kg body weight of gadobenate dimeglumine using a contrast injector at 1.5–1.8 mL/s, followed by a saline flush at 2 mL/s. MRI protocol parameters have been specified in Table 1.

Following imaging parameters were analyzed:

- iITMP [pictorial depiction in Figure 2] was developed by characterizing inner tumor margins on thin (1.5 mm section thickness) CE FSPGR sequence into
  Type 1 (lobulated)/Type 2 (irregular)/Type 3 (Ill-defined/Infiltrative) iITMP, based on the MRI analysis of the eligible patients in this study. iITMP types 1–3 were developed and evaluated based on a review of the same 224 cases included in the study population, with the radiologists blinded to the corresponding pathological findings.
- iDOI measured on thin (1.5 mm section thickness) CE FSPGR sequence and largest dimension in either coronal or axial plane was recorded in mm after excluding Peritumoral hyperenhancement (PH) [whenever present].
- Imaging T category (tumor size measured on thin CE FSPGR sequence): iT1/iT2/iT3/iT4a

### 2.3. Post-Operative Pathological Variables

Pathological variables such as WPOI, histopathological grade/type (well-differentiated/moderately differentiated/poorly differentiated), pDOI, pT category, pathologically metastatic nodes (present/absent), PNI and ENE were noted from the post-operative HPR available on EMR. WPOI was classified into five types (types 1–5) as per the established standards [4]. PNI was documented based on its presence, location (intratumoral or extratumoral), extent (unifocal or multifocal), PNI density, and caliber of the largest involved nerve [21,22]. The pathologist was blinded and had no access to the MRI findings.

### 2.4. Statistical Methods

All statistical analysis was performed using SPSS version 25 (IBM, New York, NY, USA). A significant level of *p* < 0.05 was used. Descriptive analysis was conducted for demographic data such as sex, and continuous variables such as, age, were expressed as the mean ± standard deviation (SD) or as median (Interquartile range [IQR]) based on normality of the data. Significance of correlation between categorical variables (iITMP vs. WPOI, iT vs. pT) was assessed using Kappa value, whereas Spearman test was used to evaluate correlation between continuous variables (iDOI vs. pDOI). Association between categorical variables (iITMP with WPOI, histopathological grade/type, pT category, PNI, pathologically metastatic nodes and ENE, iT with PNI and ENE, and histopathological grade with PNI) were assessed using Fisher’s Exact Test and Pearson’s Chi-squared test as applicable. 

Univariate logistic regression was performed to assess the association of iITMP and iT with PNI, and variables with *p* < 0.05 were included in the multivariable analysis.

## 3. Results

### 3.1. Demographic Characteristics

The study comprised 182 male (81%) and 42 female (19%) patients. The mean age of all patients was 47 years ± 11 SD and the median age was 46 years (IQR 39–55 IQR).

### 3.2. Correlation Between Imaging and Histopathological Parameters

- iDOI ranged from 1.9 mm to 30 mm, with a mean of 9.41 mm ± 4.74 SD. A strong positive correlation was found between iDOI and pDOI with Spearman’s rho value of 0.885 and a *p* value of <0.001. Figure 3 shows measurement of iDOI by excluding the PH.
- There were no patients with WPOI 1 and 2, so the correlation was essentially evaluated between iITMP 1, 2, 3 and WPOI 3, 4, 5. There was a substantial level of agreement between iITMP and WPOI with a Kappa value of 0.620 and a *p* value of <0.001 confirms that this agreement was statistically significant (Table 2). The most accurate match was seen between iITMP 2 and WPOI 4. Types of iITMP on MRI are shown in Figure 4.
- There were no patients in the iT4b/pT4b category. A substantial agreement was seen between iT and pT categories (Table 3) with a Kappa value of 0.758 and a *p* value of <0.001 confirms that this agreement was statistically significant.
- Category-wise analysis revealed maximum sensitivity (86.36%) and positive predictive value (PPV) [88.37%] for predicting pT1 category, maximum specificity (99.03%), negative predictive value (NPV) [97.14%] and accuracy (96.43%) for predicting pT4a category (Table 4, Table 5, Table 6 and Table 7).

### 3.3. Association Between Variables

There was a significant association (*p* = 0.001) between iITMP and pT categories (Table 8). There was significant association of iITMP and iT with PNI having a *p* value of 0.016 and <0.001 respectively (Table 9). No significant associations were observed between tumor histopathological grade and PNI or between iITMP and tumor histopathological grade.

- Univariable logistic regression analysis was also performed to evaluate the association between iITMP, iT, and PNI (Table 10). Tumors with irregular (iITMP 2) and ill-defined/infiltrative inner margins (iITMP 3) showed significantly higher Odds of PNI compared with iITMP 1 (Table 10). For iT categories, with iT1 as the reference category, the odds of PNI were significantly higher in iT2, iT3, and iT4a (Table 10).
- Both iITMP and iT demonstrated an association with PNI in univariable analysis and were subsequently included in the multivariable logistic regression model. (Table 11) On multivariate analysis, however, only iT showed significant association with PNI, and association of iITMP with PNI was not statistically significant (Table 11).
- Significant association (<0.001) of iT was also seen with ENE (Table 12). Association of iITMP with metastatic nodal involvement and ENE did not reveal any significant result.

## 4. Discussion

This study is the first to evaluate the correlation between iITMP and WPOI. The absence of WPOI 1 or 2 in our study is due to referral/selection bias inherent to a tertiary cancer center where patients present with more aggressive or advanced disease. iITMP significantly correlated with WPOI and showed significant association with pT categories. iITMP 2 and 3 showed significant association with PNI in univariate analysis but iT category remained the sole independent predictor on multivariate analysis. A strong positive correlation was found between iDOI and pDOI and a substantial agreement was seen between iT and pT categories in our study. iT showed significant association with ENE as well.

Similar to the study by Kawaguchi et al. [11], we measured iDOI by excluding the PH, and it strongly correlated with pDOI. Significant correlation between iDOI and pDOI was also seen in the studies by Tang et al. [23], Waech et al. [24], Murakami et al. [25] and Lee et al. [26]. Significant correlation between iT and pT categories were seen in Kawaguchi et al. [11], similar to our study. In the study by Sultania et al. [10], there was a fair agreement between radiological and pathological T categories (Kappa 0.32), whereas our study showed a strong agreement with a Kappa value of 0.758. Sultania et al. [10] did not include pT4a category which has been included in our study. In the study by Alharbi et al. [27], correlation of tumor stages using pDOI and iDOI showed a Kappa value of 0.68, whereas our study showed a higher Kappa value of 0.758. Studies have shown that high T category oral squamous cell carcinoma have increased propensity for PNI similar to our study [28,29,30]. To the best of our knowledge, no study has evaluated the association of iT with PNI and ENE. 

Jani et al. [31] in their study on 77 patients did not find significant association of tumor margins with PNI, unlike the findings in our study. Jani et al. [31] did not evaluate association of tumor margins with WPOI.

The strength of our study lies in the fact that imaging of a large homogenous cohort of oral tongue carcinoma treated with surgery in a single tertiary cancer center was compared with the histopathological gold standard to evaluate its correlation and association. The prognosis of OTSCC is generally poor and it is difficult to prognosticate the locally advanced OTSCC. Knowledge of adverse features such as iITMP on baseline MRI, and accurate staging using thin-section contrast-enhanced MRI can improve surgical planning in upfront resectable ones (accurate staging will help decide surgical margins) and help guide adjuvant management decisions. This information may also be explored in patients with unresectable, locally advanced diseases, in whom postoperative histopathological assessment is unavailable for identifying adverse features.

There are several limitations in our study. None of the patients in our study had WPOI 1 and 2. As a result, prospective validation in a larger cohort will be necessary to include such patients as well and evaluate the robustness of the developed iITMP to enhance the generalizability of the study. MRI-based radiomics studies should also be explored for the prediction of WPOI and PNI in oral tongue cancer [32]. Another limitation is that variability across scanners (GE vs. Philips) has not been taken into consideration. Lastly, interobserver agreement was not assessed in this study, which constitutes an additional limitation.

## 5. Conclusions

There was strong concordance between imaging inner tumor margin patterns (iITMP) and WPOI, along with a significant correlation between iITMP and pT categories in oral tongue squamous cell carcinoma. iT shows significant association with PNI and ENE. Incorporation of thin-section CE MRI can improve staging in oral tongue squamous cell carcinoma. 

## Figures and Tables

**Figure 1:**
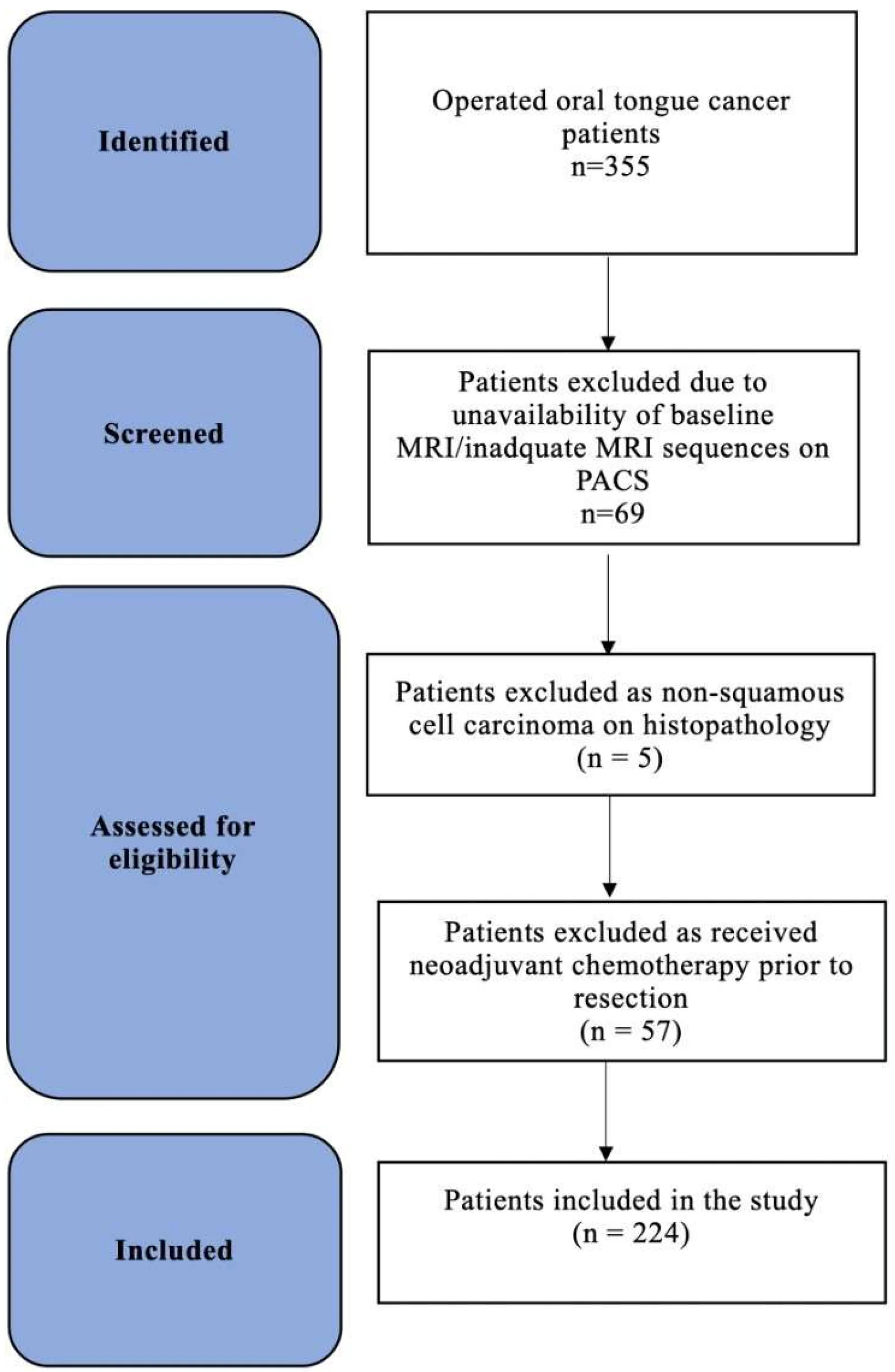
Flow diagram for selection of eligible patients.

**Figure 2:**
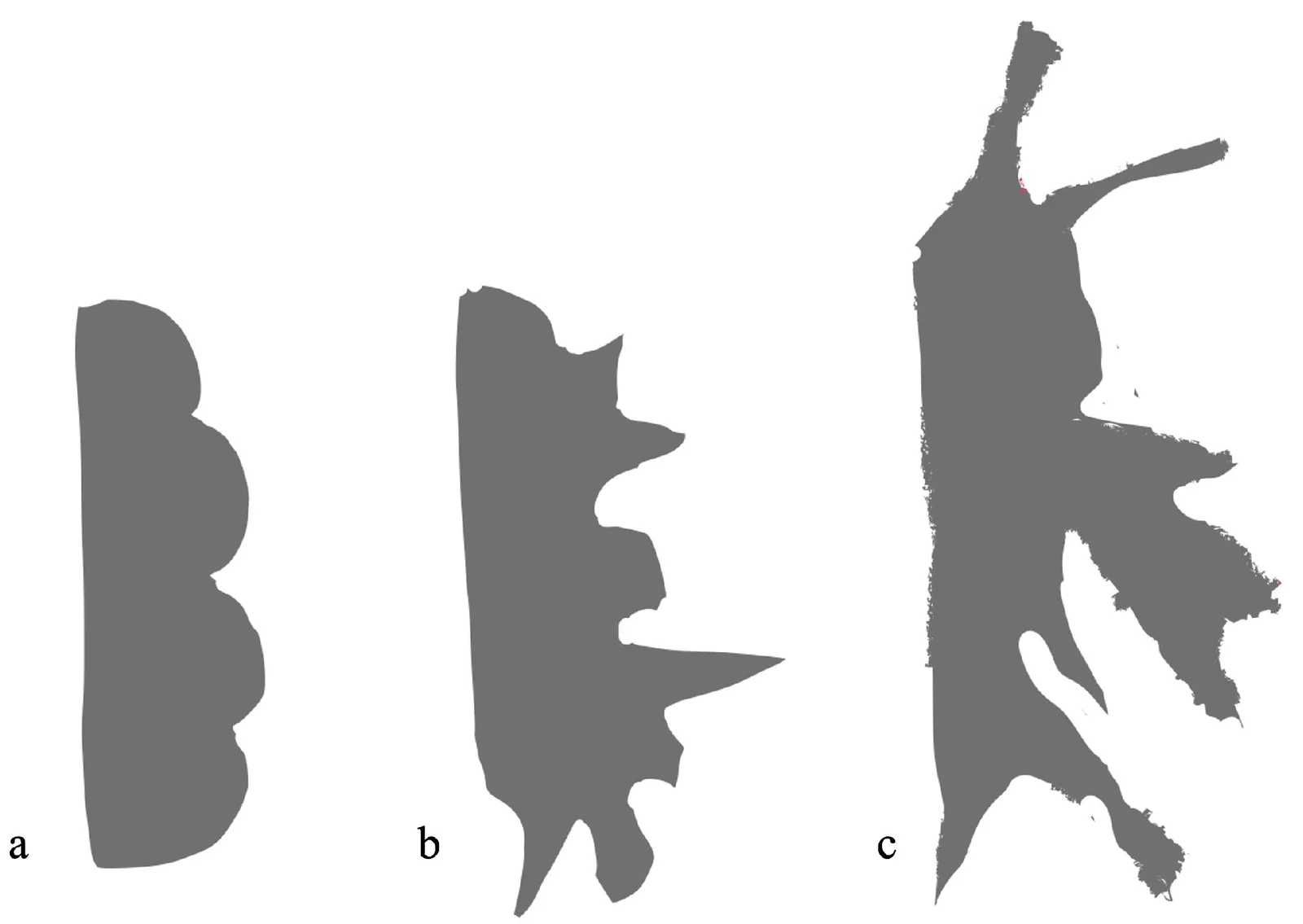
(**a**–**c**): Pictorial depiction of types of imaging Inner Tumor Margin Pattern (iITMP). (**a**) Type 1 iITMP with lobulated inner margins. (**b**) Type 2 iITMP with irregular inner margins. (**c**) Type 3 iITMP with ill-defined/infiltrative inner margins.

**Figure 3:**
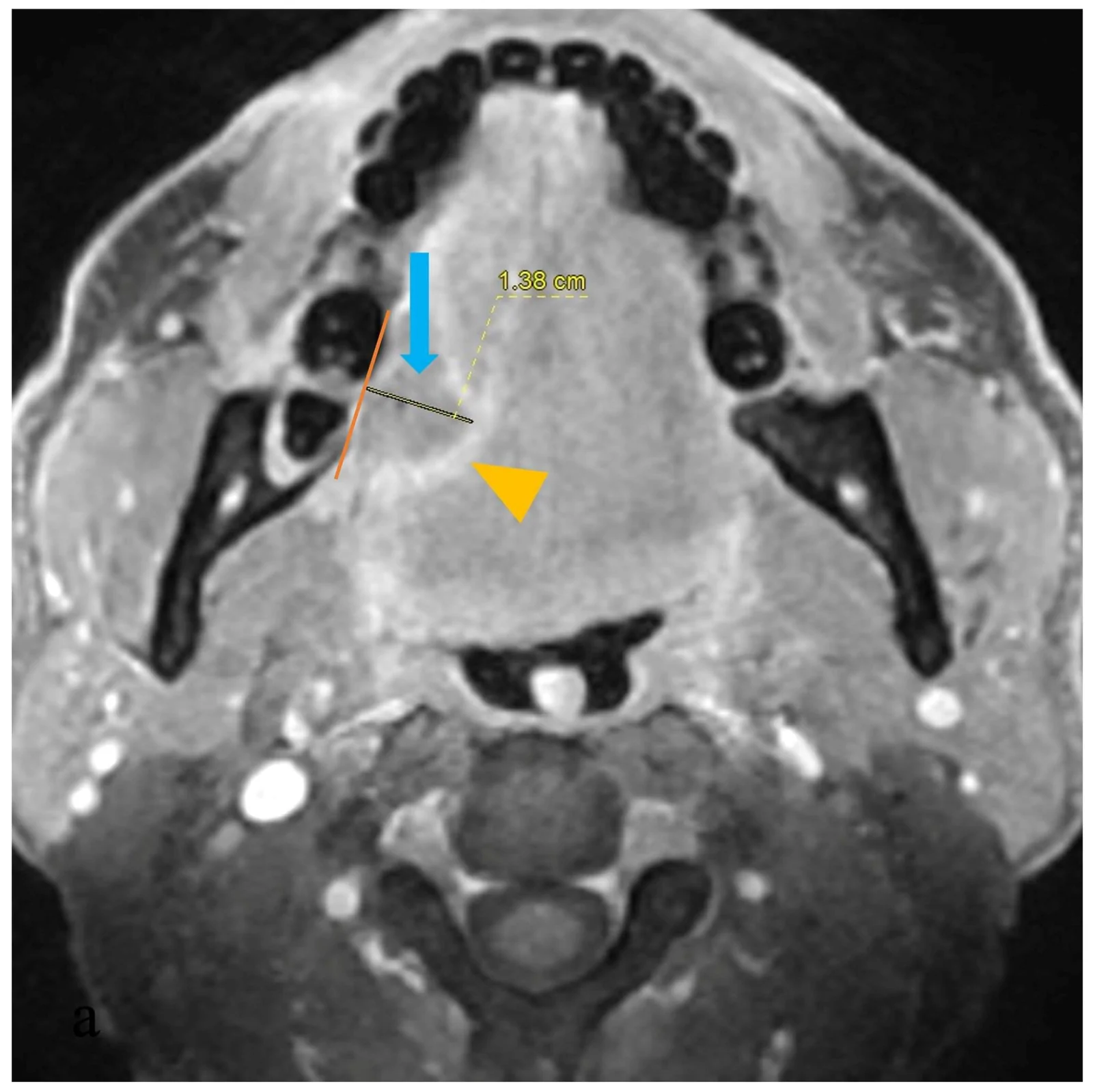
Measurement of depth of invasion (DOI) on MRI. DOI is measured from the mucosal surface (orange line) to the deepest extent of tumor. In this figure, the tumor is composed entirely of an infiltrative component, with no exophytic or ulcerative features and DOI is indicated by the arrow. The peritumoral hyperenhancement (arrowhead) is excluded from measurement of DOI.

**Figure 4:**
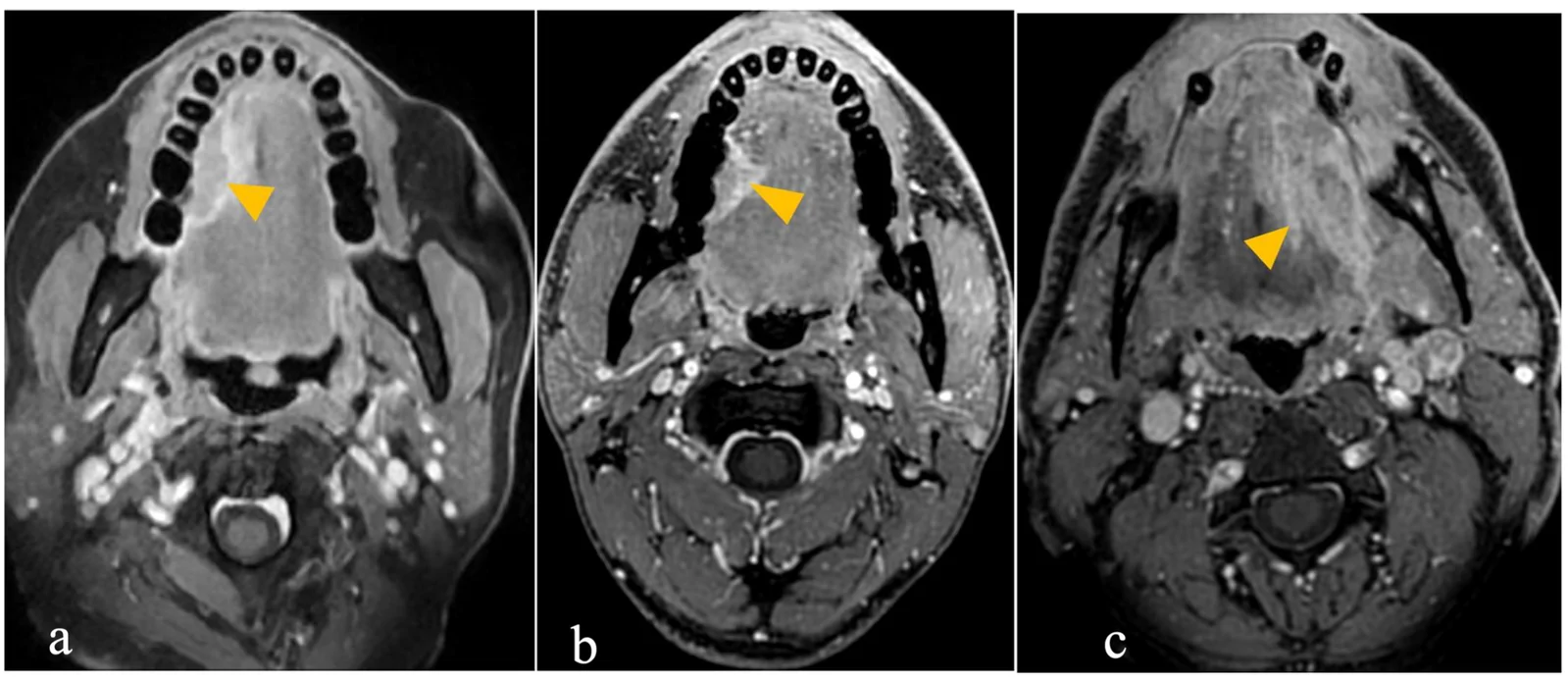
(**a**–**c**): Types of imaging Inner Tumor Margin Pattern (iITMP) on MRI. (**a**) Type 1 iITMP with lobulated inner margins (arrowhead). (**b**) Type 2 iITMP with irregular inner margins (arrowhead). (**c**) Type 3 iITMP with ill-defined/infiltrative inner margins (arrowhead).

**Table 1:** MRI protocol parameters.

**Sequences and Plane**	**Slice Thickness**	**Spacing**	**Field of View**
Coronal STIR	4 mm	1 mm	230 mm
Coronal T1WI	4 mm	1 mm	230 mm
Sagittal T2WI	4 mm	1 mm	240 mm
Axial T1WI	3 mm	0.4 mm	160 mm
Axial T2WI	3 mm	0.4 mm	160 mm
Axial T2WI	4 mm	1 mm	220 mm
DWI (b-values 0 and 800 s/mm^2^)	4 mm	1 mm	300 mm
Axial T1 post contrast fat saturated	3 mm	0.4 mm	160 mm
Coronal T1 post contrast fat saturated	4 mm	1 mm	230 mm
Axial post contrast 3D FSPGR	1.5 mm	0.75 mm	230 mm

STIR—Short tau inversion recovery, WI—Weighted Image, DWI—Diffusion Weighted Image, 3D FSPGR—Three-Dimensional Fast Spoiled Gradient Echo (FSPGR) sequences.

**Table 2:** Correlation between imaging inner tumor margin pattern (iITMP) and pathological Worst pattern of invasion.

iITMP (1/2/3) byWPOI: (3/4/5) Crosstabulation					
**Count**					
		**WPOI**			
		**3**	**4**	**5**	**Total**
**iITMP**	**1**	12	11	1	24
	**2**	3	135	14	152
	**3**	1	10	37	48
	**Total**	16	156	52	224
Symmetric Measures					
		Value	Asymptotic Standard Error ^a^	Approximate T ^b^	Approximate Significance
Measure of Agreement	Kappa	0.620	0.053	11.765	0.000
N of Valid Cases		224			

a. Not assuming the null hypothesis. b. Using the asymptotic standard error assuming the null hypothesis. iITMP: imaging Inner tumor margin patterns; WPOI: Worst pattern of invasion.

**Table 3:** Correlation between imaging primary tumor (iT) and pathological T categories.

Imaging T Category (1/2/3/4a/4b) by Pathological T Category (1/2/3/4a/4b) Crosstabulation						
**Count**						
		**Pathological T Category (1/2/3/4a/4b)**				
		**1**	**2**	**3**	**4a**	**Total**
**Imaging T category (1/2/3/4a/4b)**	**1**	38	5	0	0	43
	**2**	6	78	9	0	93
	**3**	0	9	59	6	74
	**4a**	0	1	1	12	14
	**Total**	44	93	69	18	224
**Symmetric Measures**						
		**Value**	**Asymptotic Standard Error ^a^**	**Approximate T ^b^**	**Approximate Significance**	
**Measure of Agreement**	**Kappa**	0.758	0.037	17.609	0.000	
**N of Valid Cases**		224				

a. Not assuming the null hypothesis. b. Using the asymptotic standard error assuming the null hypothesis. T: Primary tumor.

**Table 4:** Correlation of imaging primary tumor 1 category (iT1) with pathological primary tumor 1 category (pT1).

		Pathological Primary Tumor (pT1)	
		T1	Not T1
**Imaging Primary Tumor (iT1)**	T1	38	5
	Not T1	6	175
**Statistic**	**Value**	**95% CI**	
Sensitivity	86.36%	72.65% to 94.83%	
Specificity	97.22%	93.64% to 99.09%	
Positive Likelihood Ratio	31.09	13.00 to 74.38	
Negative Likelihood Ratio	0.14	0.07 to 0.30	
Disease prevalence	19.64%	14.65% to 25.46%	
Positive Predictive Value	88.37%	76.06% to 94.79%	
Negative Predictive Value	96.69%	93.27% to 98.40%	
Accuracy	95.09%	91.38% to 97.52%	

**Table 5:** Correlation of imaging primary tumor 2 category (iT2) with pathological primary tumor 2 category (pT2).

		Pathological Primary Tumor (pT2)	
		T2	Not T2
**Imaging Primary Tumor (iT2)**	T2	78	15
	Not T2	15	116
**Statistic**	**Value**	**95% CI**	
Sensitivity	83.87%	74.80% to 90.68%	
Specificity	88.55%	81.82% to 93.45%	
Positive Likelihood Ratio	7.32	4.51 to 11.89	
Negative Likelihood Ratio	0.18	0.11 to 0.29	
Disease prevalence	41.52%	34.99% to 48.27%	
Positive Predictive Value	83.87%	76.21% to 89.41%	
Negative Predictive Value	88.55%	82.89% to 92.51%	
Accuracy	86.61%	81.44% to 90.78%	

**Table 6:** Correlation of imaging primary tumor 3 category (iT3) with pathological primary tumor 3 category (pT3).

		Pathological Primary Tumor (pT3)	
		T3	Not T3
**Imaging Primary Tumor (iT3)**	T3	59	15
	Not T3	10	140
**Statistic**	**Value**	**95% CI**	
Sensitivity	85.51%	74.96% to 92.83%	
Specificity	90.32%	84.54% to 94.48%	
Positive Likelihood Ratio	8.84	5.41 to 14.43	
Negative Likelihood Ratio	0.16	0.09 to 0.29	
Disease prevalence	30.80%	24.83% to 37.30%	
Positive Predictive Value	79.73%	70.66% to 86.53%	
Negative Predictive Value	93.33%	88.73% to 96.14%	
Accuracy	88.84%	83.97% to 92.65%	

**Table 7:** Correlation of imaging primary tumor 4a category (iT4a) with pathological primary tumor 4a category (pT4a).

		Pathological Primary Tumor (pT4a)	
		T4a	Not T4a
**Imaging Primary Tumor (iT4a)**	T4a	12	2
	Not T4a	6	204
**Statistic**	**Value**	**95% CI**	
Sensitivity	66.67%	40.99% to 86.66%	
Specificity	99.03%	96.54% to 99.88%	
Positive Likelihood Ratio	68.67	16.64 to 283.33	
Negative Likelihood Ratio	0.34	0.18 to 0.65	
Disease prevalence	8.04%	4.83% to 12.40%	
Positive Predictive Value	85.71%	59.25% to 96.12%	
Negative Predictive Value	97.14%	94.65% to 98.49%	
Accuracy	96.43%	93.08% to 98.45%	

**Table 8:** Association of imaging inner tumor margin patterns (iITMP) with pathological primary tumor (pT).

iITMP (1/2/3) by Pathological T category (1/2/3/4a) Crosstabulation							*p*-Value
**Count**							
		**Pathological T Category (1/2/3/4a)**					
		**1**	**2**	**3**	**4a**	**Total**	**0.001**
**Imaging Inner Tumor Margin Patterns (1/2/3)**	**1**	13 (29.5%)	8 (8.6%)	3 (4.3%)	0	24	
	**2**	28 (63.6%)	62 (66.7%)	50 (72.5%)	12 (66.7%)	152	
	**3**	3 (6.8%)	23 (24.7%)	16 (23.2%)	6 (33.3%)	48	
	**Total**	44	93	69	18	224	

iITMP: imaging Inner tumor margin patterns; T: Primary tumor. Significant *p* values in the table have been highlighted in bold.

**Table 9:** Association of imaging inner tumor margin patterns (iITMP) and imaging primary tumor categories with perineural invasion.

	PNI			
**Variable**	**Overall, ** ***N*** ** = 224 ^1^**	**Absent, *N* = 132 ^1^**	**Present, ** ***N*** ** = 92 ^1^**	***p*** **-Value ^2^**
**iITMP_types**				**0.016**
1	24 (10.7%)	20 (15.2%)	4 (4.3%)	
2	152 (67.9%)	89 (67.4%)	63 (68.5%)	
3	48 (21.4%)	23 (17.4%)	25 (27.2%)	
**iT category**				**<0.001**
1	43 (19%)	37 (28%)	6 (6.5%)	
2	93 (42%)	63 (48%)	30 (33%)	
3	74 (33%)	30 (23%)	44 (48%)	
4a	14 (6.3%)	2 (1.5%)	12 (13%)	

^1^ *N* (%); ^2^ Fisher’s exact test; Pearson’s Chi-squared test; iITMP: imaging inner tumor margin patterns, iT: imaging primary tumor, PNI: perineural invasion. Significant *p* values in the table have been highlighted in bold.

**Table 10:** Univariate logistic regression to evaluate the association between imaging inner tumor margin patterns (iITMP) and imaging primary tumor categories (iT) with perineural invasion (PNI).

Variable	Frequency	Odds Ratio (95% CI)	*p*-Value
**iITMP types**			
1	24	1 (Reference)	
2	152	3.54 (1.15–10.86)	**0.027**
3	48	5.43 (1.61–18.29)	**0.006**
**iT category**			
1	43	1 (Reference)	
2	93	2.94 (1.12–7.72)	**0.029**
3	74	9.04 (3.40–24.09)	**<0.001**
4a	14	37.00 (6.58–208.22)	**<0.001**

iITMP: imaging inner tumor margin patterns, iT: imaging primary tumor. Significant *p* values in the table have been highlighted in bold.

**Table 11:** Multivariate logistic regression to evaluate the association between imaging inner tumor margin patterns (iITMP) and imaging primary tumor categories (iT) with perineural invasion (PNI).

Variable	Frequency	Odds Ratio (95% CI)	*p*-Value
**iITMP types**			
1	24	1 (Reference)	
2	152	1.54 (0.47–5.05)	0.478
3	48	2.11 (0.58–7.70)	0.257
**iT category**			
1	43	1 (Reference)	
2	93	2.62 (0.98–7.02)	0.055
3	74	7.77 (2.81–21.46)	**<0.001**
4a	14	30.73 (5.32–177.32)	**<0.001**

iITMP: imaging inner tumor margin patterns, iT: imaging primary tumor. Significant *p* values in the table have been highlighted in bold.

**Table 12:** Association of primary tumor on imaging (iT) with extranodal extension (ENE).

	ENE			
Variable	Overall, *N* = 224 ^1^	Absent, *N* = 165 ^1^	Present, *N* = 59 ^1^	*p*-Value ^2^
**iT_category**				**<0.001**
1	43 (19%)	39 (24%)	4 (6.8%)	
2	93 (42%)	76 (46%)	17 (29%)	
3	74 (33%)	48 (29%)	26 (44%)	
4a	14 (6.3%)	2 (1.2%)	12 (20%)	

^1^ *N* (%); ^2^ Fisher’s exact test; Pearson’s Chi-squared test; iT: imaging primary tumor; ENE: extranodal extension. Significant *p* values in the table have been highlighted in bold.

## Data Availability

Data generated or analyzed during the study are available from the corresponding author by request.

## Abbreviations

The following abbreviations are used in this manuscript:

OTSCC	Oral tongue squamous cell carcinoma
iITMP	Imaging inner tumor margin patterns
WPOI	Worst pattern of invasion
pT	Pathological primary tumor category
pN	Pathological nodal category
iT	Imaging primary tumor category
DOI	Depth of invasion
iDOI	Imaging depth of invasion
pDOI	Pathological depth of invasion
PNI	Perineural invasion
ENE	Extranodal extension
MRI	Magnetic resonance imaging
STIR	Short tau inversion recovery
CE	Contrast-enhanced
FSPGR	Fast spoiled gradient echo
PACS	Picture archiving and communication system
EMR	Electronic medical record
HPR	Histopathology report
GE	General Electric
SPSS	Statistical Package for the Social Sciences
SD	Standard deviation
IQR	Interquartile range
PPV	Positive predictive value
NPV	Negative predictive value
PH	Peritumoral hyperenhancement
OS	Overall survival
DFS	Disease-free survival
DSS	Disease-specific survival
CRT	Chemoradiotherapy
UICC	Union for International Cancer Control
TNM	Tumor, Node, Metastasis
CI	Confidence interval
